# Diffuse Brain Injury Induces Acute Post-Traumatic Sleep

**DOI:** 10.1371/journal.pone.0082507

**Published:** 2014-01-08

**Authors:** Rachel K. Rowe, Martin Striz, Adam D. Bachstetter, Linda J. Van Eldik, Kevin D. Donohue, Bruce F. O'Hara, Jonathan Lifshitz

**Affiliations:** 1 Barrow Neurological Institute at Phoenix Children's Hospital, Phoenix, Arizona, United States of America; 2 Department of Child Health, University of Arizona College of Medicine–Phoenix, Phoenix, Arizona, United States of America; 3 Phoenix Veteran Affairs Healthcare System, Phoenix, Arizona, United States of America; 4 Department of Anatomy and Neurobiology, College of Medicine, University of Kentucky, Lexington, Kentucky, United States of America; 5 Department of Biology, College of Arts and Sciences, University of Kentucky, Lexington, Kentucky, United States of America; 6 Department of Electrical and Computer Engineering, College of Engineering, University of Kentucky, Lexington, Kentucky, United States of America; 7 Department of Physical Medicine & Rehabilitation, University of Kentucky College of Medicine, Lexington, Kentucky, United States of America; 8 Spinal Cord and Brain Injury Research Center (SCoBIRC), University of Kentucky College of Medicine, Lexington, Kentucky, United States of America; 9 Sanders-Brown Center on Aging, University of Kentucky College of Medicine, Lexington, Kentucky, United States of America; Hospital Nacional de Parapléjicos – SESCAM, Spain

## Abstract

**Objective:**

Clinical observations report excessive sleepiness immediately following traumatic brain injury (TBI); however, there is a lack of experimental evidence to support or refute the benefit of sleep following a brain injury. The aim of this study is to investigate acute post-traumatic sleep.

**Methods:**

Sham, mild or moderate diffuse TBI was induced by midline fluid percussion injury (mFPI) in male C57BL/6J mice at 9:00 or 21:00 to evaluate injury-induced sleep behavior at sleep and wake onset, respectively. Sleep profiles were measured post-injury using a non-invasive, piezoelectric cage system. In separate cohorts of mice, inflammatory cytokines in the neocortex were quantified by immunoassay, and microglial activation was visualized by immunohistochemistry.

**Results:**

Immediately after diffuse TBI, quantitative measures of sleep were characterized by a significant increase in sleep (>50%) for the first 6 hours post-injury, resulting from increases in sleep bout length, compared to sham. Acute post-traumatic sleep increased significantly independent of injury severity and time of injury (9:00 vs 21:00). The pro-inflammatory cytokine IL-1β increased in brain-injured mice compared to sham over the first 9 hours post-injury. Iba-1 positive microglia were evident in brain-injured cortex at 6 hours post-injury.

**Conclusion:**

Post-traumatic sleep occurs for up to 6 hours after diffuse brain injury in the mouse regardless of injury severity or time of day. The temporal profile of secondary injury cascades may be driving the significant increase in post-traumatic sleep and contribute to the natural course of recovery through cellular repair.

## Introduction

Traumatic brain injury (TBI) is a major cause of death and disability throughout the world with little pharmacological treatment for the individuals who suffer from lifelong neurological morbidities associated with TBI. Brain injury can lead to both short and long-term impairment, including cognitive [1], and behavioral [2] deficits as well as increasing the risk for developing neurodegenerative disease [3] and/or psychiatric disorders [4]. Little can be done to mitigate the mechanical disruption associated with the primary insult and the biochemical cascades initiated shortly after the time of injury can impair physiological function and ultimately worsen long-term outcome [5].

Clinical studies have provided evidence to support the hypothesis that brain injury contributes to chronic sleep disturbances as well as leads to excessive daytime sleepiness [6]–[9]. Far less is known about the acute relationship between TBI and sleep. Immediately after TBI, secondary injury mechanisms may impair physiological functions associated with the homeostatic regulation of sleep. For example, secondary injury processes result in glia activation and initiation of marked inflammatory responses. Injury-induced inflammation is mediated by the production of cytokines, such as pro-inflammatory interleukin-1 beta (IL-1β), which can have dual roles as sleep regulatory substances (SRSs) [10], [11]. Elevated cytokine signaling has been observed across experimental models and human TBI, highlighting their involvement in pathological and reparative processes triggered by injury [12]–[15]. However, cytokines which are SRSs can also modulate sleep-wake behavior, primarily enhancing sleep by acting on sleep circuits of the brain [11], [16].

Secondary injury mechanisms of TBI deplete adenosine-5′-triphosphate (ATP) causing failure of energy-dependent membrane ion pumps, increase reactive oxygen species (ROS), increase intracellular concentrations of free radicals caused by activation of lipid peroxidases, as well as increase inflammatory mediating cytokines [17], [18]. A low energy state, high ROS, and the increase of certain cytokines have all been implicated in increased sleep propensity and sleep duration [19], [20]. Thus, secondary injury processes following TBI have the potential to induce post-traumatic sleep. The magnitude and duration of the induction of post-traumatic sleep are unknown. Further, as these cellular processes continue, chronic sleep issues may develop.

The biological function of sleep remains controversial, however, prevailing hypotheses suggest the function of sleep is restorative, conservative, and adaptive [21], [22]. In the absence of sleep, humans exhibit deficits in attention, memory, learning, and higher cognitive processes [23]. Sleep is regulated by homeostatic processes as well as circadian processes [24] such that injury may disrupt the signaling required to maintain a healthy sleep profile. If sleep is regenerative in function, then acute post-traumatic sleep may improve outcome from brain injury. This study is the first of its kind to investigate acute sleep following diffuse brain injury.

To characterize acute sleep patterns in brain-injured mice, a non-invasive sleep monitoring cage system was used to continuously record post-traumatic sleep. The sleep monitoring cage system allows reliable, immediate and continuous sleep monitoring by using piezoelectric materials configured as highly-sensitive pressure detectors incorporated into the bottom of the animal cage to determine rest-activity based on motion and breathing patterns [25]. Sleep is discriminated from wake every 2 seconds from tapered 8 second overlapping windows based on classification algorithms that exploit the limited mouse sleeping postures and distinct respiratory patterns consistent with sleep, compared to rest and activity [25], [26]. This technology is also adapted to measure the polycyclic sleep pattern of mice, accounting for short interruptions and brief arousals in sleep. The cage system recognizes short bouts (a single sleep episode) lasting only seconds as well as extended bouts lasting longer than a minute.

Sleep can complicate the understanding of injury processes following TBI [9]. The investigation of post-traumatic sleep may lead to rational interventions to mitigate damage. The present study was designed to examine injury-induced alterations in acute sleep following TBI. Clinical observations indicate that patients report excessive sleepiness immediately following TBI [6]. In view of these observations, we hypothesized that diffuse brain injury would induce acute post-traumatic sleep in the mouse. The lack of biomedical research surrounding the controversial question whether one should sleep or be frequently awoken immediately following brain injury adds to the importance of investigating post-traumatic sleep, specifically in the acute period. In neurosurgical wards TBI patients are frequently awoken during the first day after injury to check for possible worsening of their consciousness, a condition that requires immediate action. In more mild conditions, the practice of keeping a brain-injured individual awake is controversial. Here we demonstrate significant increases in acute sleep post-injury regardless of injury severity or time of day. Post-traumatic sleep occurs during the same time as increased cortical expression of SRS cytokines and inflammation.

## Methods

### Animals and Ethics Statement

Male C57BL/6J mice (Harlan Laboratories, Inc., Indianapolis, IN) were used for all experiments (n = 75). The animals were housed in a 14 h light/10 h dark cycle at a constant temperature (23°C±2°C) with food and water available ad libitum according to the Association for Assessment and Accreditation of Laboratory Animal Care International. Animals were acclimated to their environment following shipment for at least three days prior to any experiments. After surgery, animals were evaluated daily for post-operative care by a physical examination and documentation of each animal's condition. All studies were approved by the University of Kentucky Institutional Animal Care and Use Committee (IACUC Protocol Number: 2007-0142). All surgery was performed under isoflurane anesthesia, and efforts were made to minimize suffering.

### Midline Fluid Percussion Injury (mFPI)

Adult male C57BL/6J mice (20–24 g) were subjected to midline fluid percussion injury (mFPI) consistent with methods previously described [27]. Animal numbers are indicated in the results section and figure legends for individual studies. Mice were anesthetized using 5% isoflurane in 100% oxygen for five minutes and the head of the animal was placed in a stereotaxic frame with continuously delivered isoflurane at 2.5% via nosecone. While anesthetized, the animal's body temperature was maintained using a Deltaphase® isothermal heating pad (Braintree Scientific Inc., Braintree, MA). A midline incision was made exposing bregma and lambda, and fascia was removed from the surface of the skull. A trephine (3 mm outer diameter) was used for the craniotomy, centered on the sagittal suture between bregma and lambda without disruption of the dura. An injury cap prepared from the female portion of a Luer-Loc needle hub was fixed over the craniotomy using cyanoacrylate gel and methyl-methacrylate (Hygenic Corp., Akron, OH). The incision was sutured at the anterior and posterior edges and topical Lidocaine ointment was applied. The injury cap was closed using a Luer-Loc cap and animals were placed in a heated recovery cage and monitored until ambulatory before being returned to their sleep cage.

For injury induction 24 hours post-surgery, animals were re-anesthetized with 5% isoflurane delivered for five minutes. The cap was removed from the injury-hub assembly and the craniotomy was visually inspected through the hub. The hub was then filled with normal saline and attached to the male end of the fluid percussion device (Custom Design and Fabrication, Virginia Commonwealth University, Richmond, VA). An injury of moderate severity (1.2–1.3 atm), mild severity (0.8 atm) or sham injury was administered by releasing the pendulum onto the fluid-filled cylinder. Sham-injured animals underwent the same procedure except the pendulum was not released. Animals were monitored for the presence of a forearm fencing response, and righting reflex times were recorded for the injured animals as indicators of injury severity [28]. The righting reflex time is the total time from the initial impact until the animal spontaneously rights itself. The fencing response is a tonic posturing characterized by extension and flexion of opposite arms that has been validated as an overt indicator of injury force magnitude [28]. The injury hub was removed and the brain was inspected for uniform herniation and integrity of the dura. Animals in which the dura was compromised were excluded from all studies as technical failures. The incision was cleaned using saline and closed using sutures. Moderate brain-injured animals had righting reflex recovery times greater than six minutes and a positive fencing response, and mild injured animals had righting reflex times between two and four minutes and no fencing response. Sham injured animals recovered within 10 seconds. After spontaneously righting, animals were placed in a heated recovery cage and monitored until ambulatory before being returned to their sleep cage (approximately 5 to 15 minutes). Adequate measures were taken to minimize pain or discomfort.

### Sleep Recordings

The non-invasive sleep cage system (Signal Solutions, Lexington, KY) used in this study consisted of 16 separate units that could simultaneously monitor the sleep and wake states over several days. The cage system classified sleep and wake behavior based on methods previously described [25], [26]. Each cage unit housed the mice individually with separate 7×7 inch walled compartments and attached food and water structures [25]. The cages had open bottoms that allowed them to be placed on a base with a Polyvinylidine Difluoride (PVDF) sensor on the cage floor [25]. The PVDF sensors were coupled to an input differential amplifier and pressure signals were generated and classified by the non-invasive high-throughput classifier as motions consistent with either wake activity or the inactivity associated with sleep [25]. Sleep was characterized primarily by periodic pressure measurements with regular amplitudes, typical of respiration from a still mouse. In contrast, movements characteristic of wake were both the absence of the characteristic sleep signal and higher amplitude, irregular spiking associated with volitional movements. The piezoelectric signals were classified by the automated sleep scoring system in two second epochs as “sleep” or “wake”. The tapered segmentation window was advanced every two seconds and features associated with the characteristics just described were computed. A linear discriminant classifier based on these features was applied to assign a binary label given to each point associated with the center of the window [25]. Data collected from the cage system were binned over specified time periods (e.g. 5 minutes, 1 hour) using a rolling average of the percent sleep, as well as binned by length of individual bouts of sleep and the median bout lengths were calculated.

**Figure 1:**
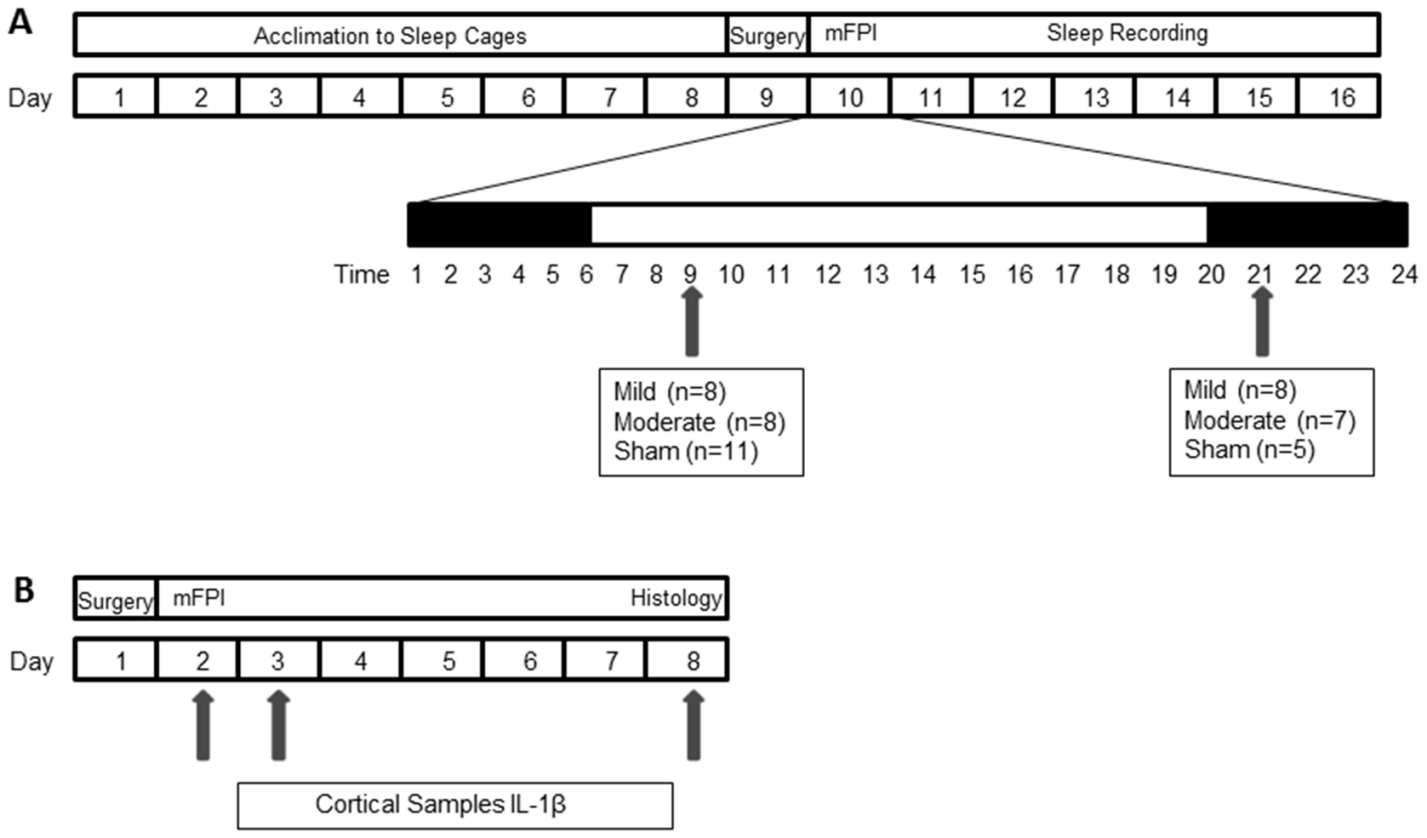
Schematic of the study design. Two cohorts of mice were used based on experimental outcome measures: (**A**) sleep recordings and (**B**) cortical samples and histology. (**A**) Mice were acclimated to piezoelectric sleep cages for 8 days while sample sleep recordings were monitored to test signal integrity. All mice received a midline craniotomy one day prior to brain or sham injury. Mice were divided into 2 groups based on the time of day they were subjected to injury (9:00, 21:00). Within each group, mice were selected at random and subjected to sham, mild (0.8 atm) or moderate (1.2–1.3 atm) diffuse brain injury by midline fluid percussion (mFPI) (n = 47). Following injury, mice were placed back into piezoelectric sleep cages and post-traumatic sleep was recorded for 7 days. (**B**) For biochemistry and histology, mice received a midline craniotomy one day prior to injury or sham injury. Mice were subjected to sham, or moderate (1.2–1.3 atm) diffuse brain injury (9:00) and cortical samples were retrieved at 1, 3, 9, 12, 24, 48, 168 hrs (n = 25). Tissue was also collected and prepared for histology 6 hrs post-injury (n = 3).

Mice were acclimated to the cages and sensors were tested for 8 days prior to injury (Figure 1A). The animals were removed from their home cages for the midline craniotomy surgery and were placed back into their specific cage following the surgery. The following day the mice were removed from their home cage and were subjected to mFPI or sham injury. As soon as the mice were ambulatory (approximately 5 to 15 minutes), they were returned to their original sleep cage and the sleep recordings started to measure sleep continuously for 7 days [25]. Previous validation of the sleep cages in agreement with human observation resulted in classification rates of 90% or higher [25]. This sleep cage system was a valid method for monitoring acute sleep in injured animals without confounding the injury. Placement of invasive EEG equipment for recordings can compromise the dura and adds external weight to the head of the rodent possibly exacerbating the brain-injury.

A multivariate analysis of variance (ANOVA) was used to compare the mean percent sleep of brain-injured mice to uninjured shams over time. Statistical significance was assigned when p<0.05.

### Tissue preparation and cytokine measurement

At selected time points (1, 3, 9, 12, 24, 48, 168 hours) post-injury or sham operation, mice were given an overdose of sodium pentobarbital and transcardially perfused with phosphate buffered saline (PBS) (Figure 1A). Mice were decapitated and the brains were dissected on ice and snap frozen in liquid nitrogen then stored at −80°C until used. The protein levels of a panel of inflammatory cytokines were measured in the neocortex by Meso Scale Discovery (MSD) multiplex immunoassay (sector imager 2400, Meso Scale Discovery; Gaithersburg Maryland) (Figure 1B) as previously described [29]. Brain cortex was homogenized using high shear homogenizer (Omni TH115), in a 1∶10 (w/v) of ice-cold lysis buffer consisting of PBS containing 1 µg/ml Leupeptin, 1 mM PMSF, and 1 mM EDTA. The cortical homogenate was centrifuged at 14,000×g for 20 minutes at 4°C in a microcentrifuge. Fifty microliters of the resulting supernatant was loaded per well of the custom MSD plate, and IL-1β levels were determined by MSD assay (Mouse Proinflammatory 7-Plex Ultra-Sensitive (K15012C)). IL-1β levels in the cortex were normalized to the total amount of protein in the sample loaded as determined by BCA Protein Assay (Pierce).

Cytokine levels of IL-1β were compared between uninjured sham and brain-injured mice. Increases in cytokine levels in the brain-injured mice were analyzed over a time course using a one-way ANOVA and selected comparisons were made using the Bonferroni post hoc test.

### Tissue preparation for immunohistochemistry

At 6 hours post-injury or sham operation, mice were given an overdose of sodium pentobarbital and transcardially perfused with 4% paraformaldehyde after a (PBS) flush. Brains were removed and placed in 4% paraformaldehyde overnight. Brains were immersed in serial dilutions (10%, 20%, and 30%) of sucrose for 24 hours each. The brains were removed from the 30% sucrose and frozen at −20°C. After freezing, brains were sectioned in the coronal plane at 20 µm, mounted onto glass slides, and stored at −80°C.

### IBA-1 immunohistochemistry

Slides were removed from −80°C, placed in an oven at 60°C for approximately 4 hours and then rinsed three times for 5 minutes each in PBS. Next, the slides were incubated in 4% goat serum blocking solution for 1 hour. The slides were incubated with the primary antibody (rabbit anti-ionized calcium binding adaptor molecule 1 (IBA-1), 1∶1000, Wako Chemicals 0199-19741) and stored at room temperature overnight. Slides were rinsed three times in PBS and the secondary antibody (biotinylated horse anti-rabbit, 1∶250, Vector Laboratories) was added then slides were incubated on a rocker at room temperature for 1 hour. The slides were washed in PBS three times for 5 minutes each and tertiary stain was applied (streptavidin Alexa© Fluor 594, 1∶1000, Jackson Laboratories) and slides were incubated for 1 hour at room temperature. Lastly, slides were rinsed three times in PBS and coverslipped with Fluoromount-G anti-fade medium (Southern Biotech). The cortex was examined for microglia activation in response to brain-injury using a Zeiss Axio Scope with attached digital camera.

## Results

### Diffuse TBI induces acute post-traumatic sleep in the mouse

**Figure 2:**
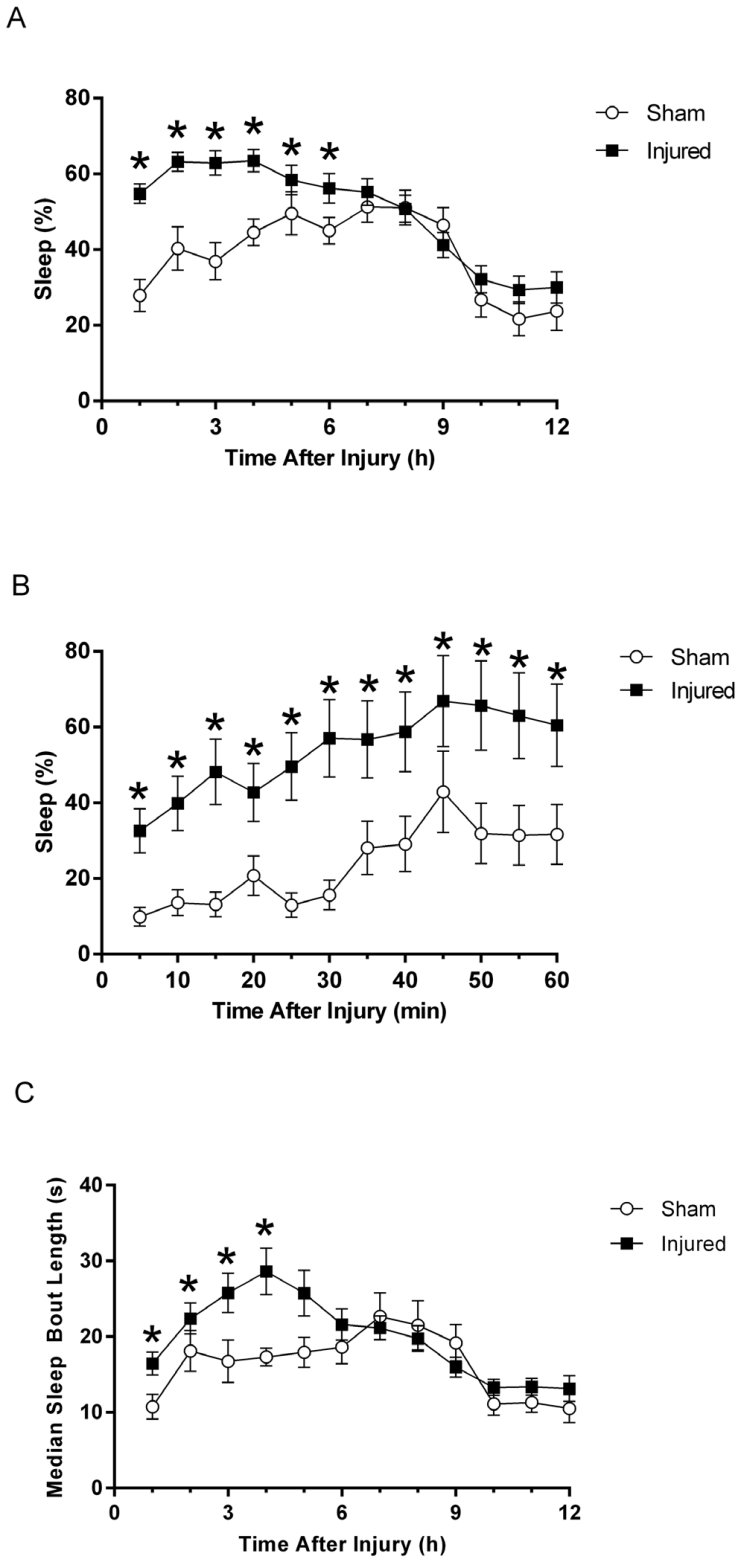
Diffuse TBI in the mouse disrupts acute post-traumatic sleep parameters compared to uninjured sham. (**A**) A multivariate ANOVA showed a significant increase in mean percent sleep over the first 6 hours post-injury compared to the uninjured sham (mean ±SEM; sham n = 16; injured n = 31; F(1, 45) = 6.545, p = 0.00007). After 6 hours post-injury, the mean percent sleep of injured mice normalized to sham mean percent sleep levels and remained comparable for 7 days post-injury (data not shown). (**B**) A detailed analysis of the acute post-traumatic sleep (in the first hour) following diffuse TBI indicated a significant time dependent effect on the increase in sleep. A multivariate ANOVA of the rolling average of the mean percent sleep over 5 min intervals showed post-traumatic sleep significantly increased over the first hour post-injury with a significant effect of time (mean ±SEM; sham n = 16; injured n = 31; F(11,495) = 8.22, p<0.0001) and group (mean ±SEM; sham n = 16; injured n = 31; F(1,45) = 37.00, p<0.0001). Bonferroni post hoc analysis was used (*, p<0.05). (**C**) Acutely post-injury, the brain-injured mice showed an increase in median bout length compared to shams. A multivariate ANOVA revealed an increase in bout length significant over the first 4 hours post-injury (mean ±SEM; sham n = 16; injured n = 31; F(1,45) = 2.9138, p = 0.032). This increase in bout length suggested that the increase in mean percent sleep observed acutely post-injury could result from mice sleeping for longer durations, as opposed to sleeping more bouts after diffuse TBI.

Immediately after diffuse TBI, mean percent sleep was significantly increased in brain-injured animals compared to sham for the first 6 hours post-injury (F(1, 45) = 6.545, p = 0.00007) (Figure 2A). After 6 hours post-injury, the mean percent sleep of the injured mice (n = 31) normalized and was indistinguishable from sleep in the sham (n = 16) through 7 days post-injury (data not shown). A more detailed analysis was performed by calculating the mean percent sleep over five minute intervals for the first hour post-injury to examine the increase in sleep observed acutely post-injury. The mean percent sleep showed a significant time-dependent increase in sleep over the first hour post-injury (F(11,495) = 8.22, p<0.0001) (Figure 2B) until maximum sleep was reached. In addition, analysis over the first hour-post injury showed a significant group effect (F(1,45) = 37.00, p<0.0001) (Figure 2B) indicating TBI induced a significant increase in mean percent sleep compared to the uninjured sham. Increase in acute post-traumatic sleep in the diffuse brain-injured mouse was associated with increased median bout lengths of sleep (Figure 2C). We observed a significant increase in the median bout length of brain-injured mice compared to sham for the first 4 hours post-injury (F(1,45) = 2.9138, p = 0.032). This increase in bout length indicated that the increase in mean percent sleep observed acutely post-injury (Figure 2A) could result from mice sleeping for longer durations during each bout, as opposed to sleeping more bouts after diffuse TBI.

### Post-traumatic sleep bouts are interrupted by volitional movement and arousal similar to uninjured shams

**Figure 3:**
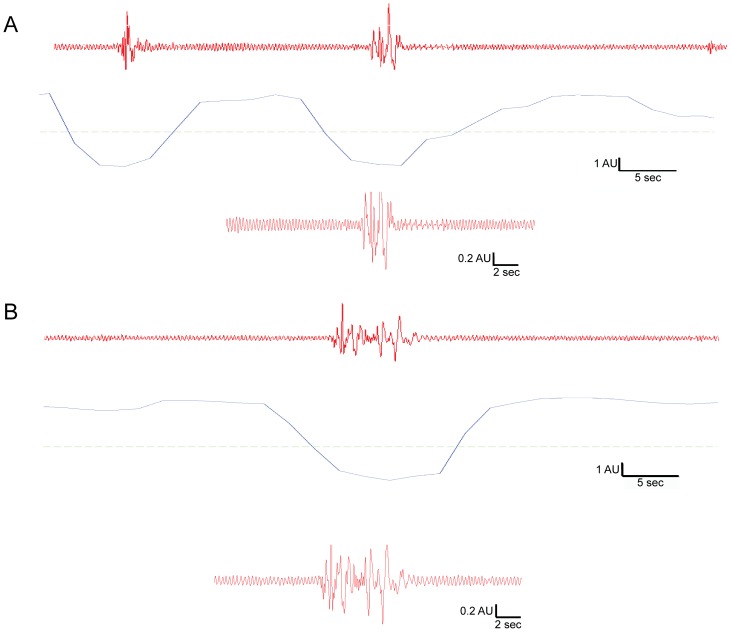
Representative sleep-wake recordings in the first hour post-injury showed sleep bouts interrupted by brief arousal and movement. Uninjured sham mice showed a periodic rhythm of breathing motion (∼3 Hz) with regular amplitude typical of sleep, interrupted by high frequency and amplitude signals corresponding to movement consistent with an awake mouse (**A**). Diffuse brain-injured mice showed similar rhythmic breathing classified as sleep interrupted by frequency and amplitude variations corresponding to movement during interbout intervals of sleep (**B**). The red lines represent the raw piezoelectric sensor data over a one minute (top) or 25 second (bottom) interval. The discontinuous blue line indicates the decision classifier over two second intervals to classify sleep activity from wake activity. The broken green line delineates the threshold (in arbitrary units) to determine sleep activity (above the threshold) from wake activity (below the threshold).

Immediately after diffuse TBI, mean percent sleep was significantly increased in brain-injured mice compared to uninjured sham. To analyze the signals used to classify sleep, the raw piezoelectric sensor data was extracted and compared between brain-injured and sham mice within the first hour post-injury. Uninjured sham mice showed a periodic rhythm associated with the motion of breathing, approximately 3 Hz, with regular amplitude typical of sleep in the mouse (Figure 3A). These sleep bouts were interrupted by higher frequency and amplitude signals that correspond to movements consistent with wake activity (Figure 3A). The sleep-wake classifier plotted with the decision threshold (Figure 3, below raw signal) were classified as sleep activity above the threshold and as wake activity below the threshold. In brain-injured mice, sleep activity showed similar rhythmic breathing classified as sleep (Figure 3B). As in the uninjured sham, sleep was interrupted by high amplitude and frequency signals corresponding to volitional movement. Interruptions of sleep bouts by volitional movement indicate the brain-injured animals terminate sleep bouts in a similar manner to uninjured mice, suggesting that brain-injured mice are responsive, capable of movement, and not in a comatose state of unresponsiveness. As shown, sleep bouts in brain-injured mice are longer in duration than in uninjured mice (Figure 3B).

### Increase in acute post-traumatic sleep in the diffuse brain-injured mouse was independent of injury time of day

**Figure 4:**
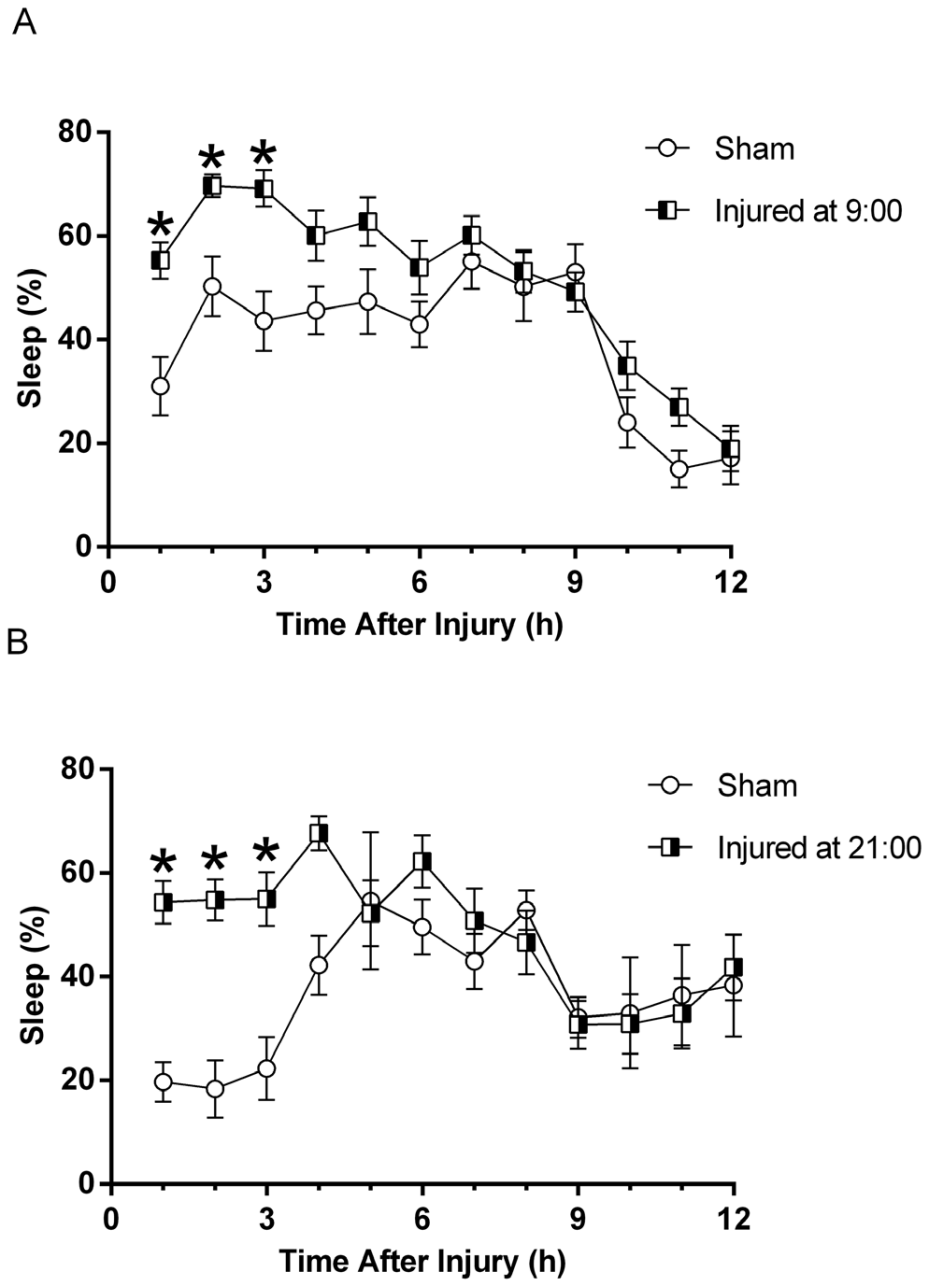
Significant increase in post-traumatic sleep is independent of the time of day of the injury. Mice subjected to mild or moderate injury at 9:00 (**A**), following the dark/light transition showed significant increases in acute post-traumatic sleep compared to uninjured sham. A multivariate ANOVA and Bonferroni post-hoc analysis was used (mean ±SEM; sham n = 12; injured n = 17; F(1,25) = 15.95); *, p<0.05). Mice subjected to mild or moderate injury at 21:00 (**B**), following the light/dark transition also showed significant increases in acute post-traumatic sleep compared to sham. A multivariate ANOVA and Bonferroni post-hoc analysis was used (mean ±SEM; sham n = 5; injured n = 14; F(1,17) = 4.42; *, p<0.05). An increase in sleep is observed acutely following TBI and is observed over the course of the first 3 hours in injured mice compared to sham. After 3 hours, sleep began to normalize in the injured animals and became indistinguishable from sleep in the sham. Mean percent sleep of uninjured sham mice in the 9:00 group was significantly higher than the mean percent sleep of sham mice in the 21:00 group (F(1,15) = 6.303, p = 0.0240), as expected.

Sham or brain injury was administered at transitional time points (9:00 or 21:00) in the light/dark cycle (Figure 1A). We observed an increase in the mean percent sleep of brain-injured mice compared to sham when mice were injured at 9:00, following the onset of the light cycle (Figure 4A). Brain-injury resulted in a significant increase in mean percent sleep for the first 3 hours following injury as compared to the mean percent sleep of sham (F(1,25) = 15.95, p = 0.0005). Similarly, we observed an increase in post-traumatic sleep when mice were subjected to injury at 21:00, following the onset of the dark cycle (Figure 4B). We recorded a significant increase in mean percent sleep for the first 3 hours following brain-injury compared to uninjured shams (F(1,17) = 4.42, p = 0.0506). Regardless of injury time of day, post-traumatic sleep was increased to comparable levels (45–65%) and became indistinguishable from sleep in the sham after 3 hours post-injury. In contrast, diurnal pressures associated with the change in the light/dark cycle were evident on the mean percent sleep of uninjured sham animals, as expected. The mean percent sleep of uninjured sham mice in the 9:00 group was significantly higher than the mean percent sleep of sham mice in the 21:00 group (F(1,15) = 6.303, p = 0.0240). This finding is representative of the nocturnal activity of mice.

### Increase in acute post-traumatic sleep in the diffuse brain-injured mouse was independent of injury severity

**Figure 5:**
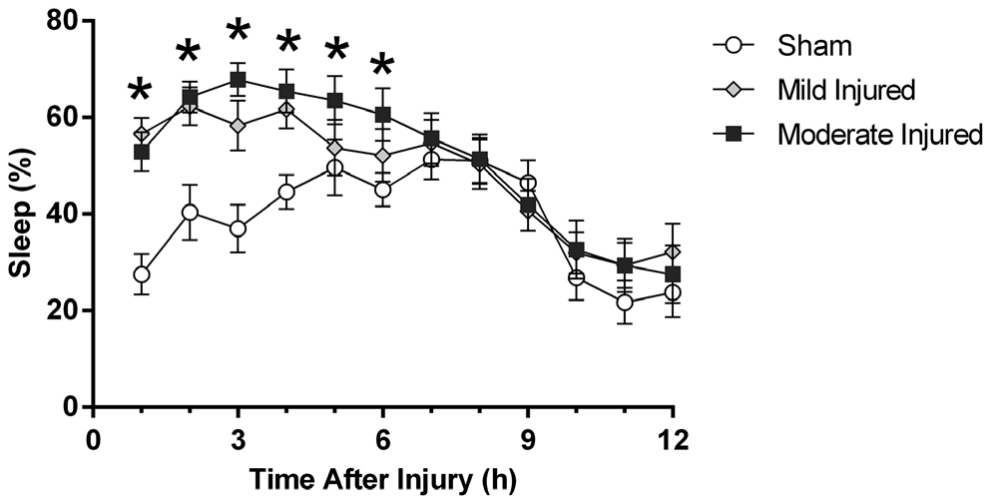
The significant increase in post-traumatic sleep is observed acutely following both mild and moderate injury. A multivariate ANOVA showed a significant increase in mean percent sleep between injured mice and uninjured shams over the first 6-injury with no significant difference between mildly injured mice compared to moderately injured mice (mean ±SEM; sham n = 16; mild n = 16; moderate n = 15; F(2,44) = 3.4773, p = 0.00037).

Two levels of experimental injury severity were used to test the effects of injury severity on post-traumatic sleep. We define injury severity as mild (0.8 atm) and moderate (1.2–1.3 atm) according to the righting-reflex and the fencing response (see methods). Post-traumatic sleep was not significantly different between mild and moderate brain-injured mice (Figure 5). A significant increase in post-traumatic sleep was observed acutely following both mild and moderate injury compared to the uninjured sham (F(2,44) = 3.4773, p = 0.00037). No significant difference was found between mild brain-injured mice and moderate brain-injured mice, indicating the significant increase in post-traumatic sleep is independent of injury severity.

### Secondary injury responses temporally associate with the increase in acute post-traumatic sleep in the diffuse brain-injured mouse

**Figure 6:**
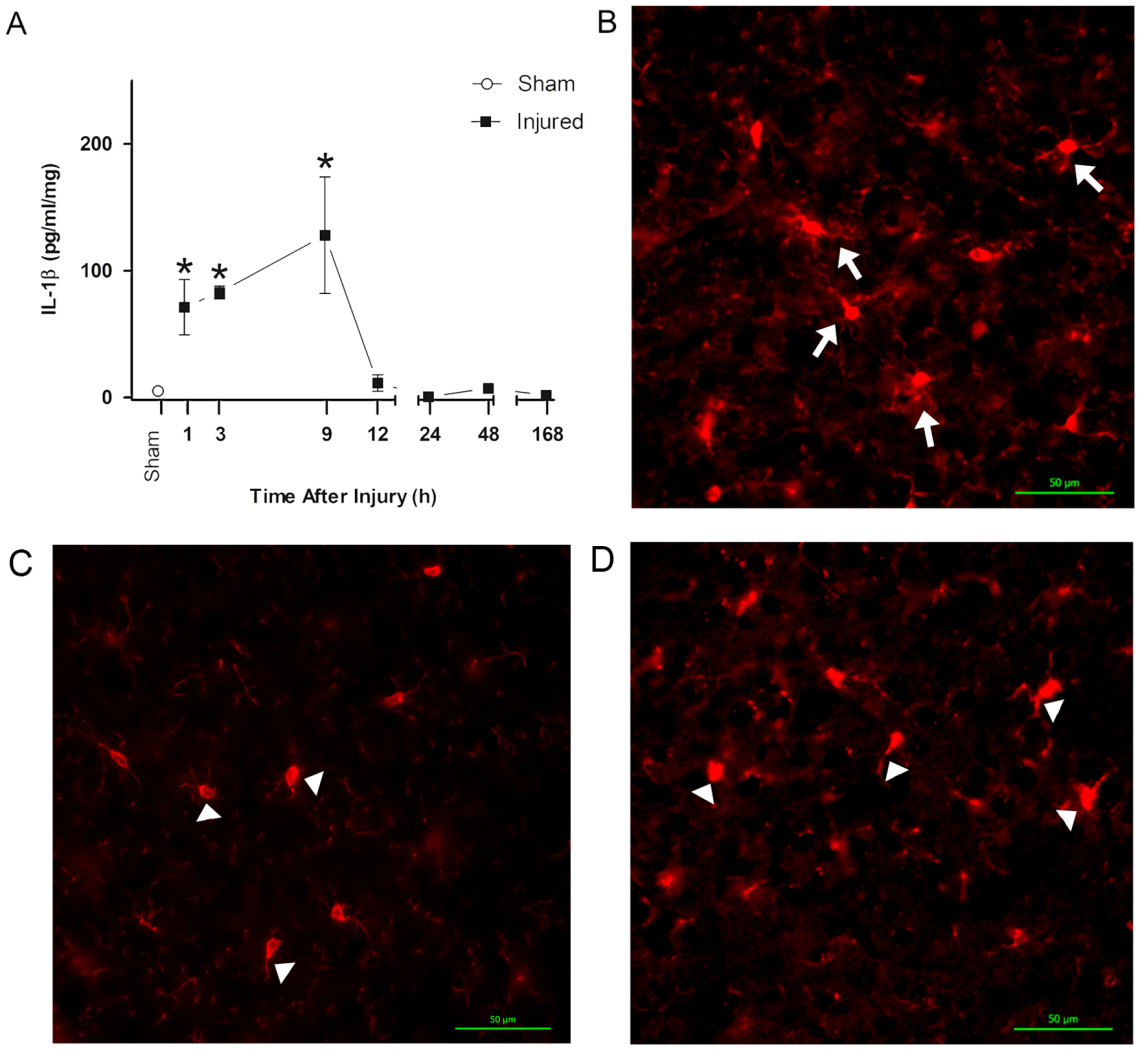
(A) Temporal profile of IL-1β. The temporal profile indicated that levels in the cortex increase rapidly following moderate injury (9:00) as compared to uninjured sham. Levels of IL-1β peak at or near 9 hours post-injury and return to baseline levels by 12 hours post-injury (One-way ANOVA, mean ±SEM; sham n = 7; injured n = 22; F(7,21) = 6.474; p = 0.0004). Selected comparisons were made (Bonferroni post-hoc), asterisk denotes significance (*, p<0.05) compared to sham. (B, C, D) Microglia morphology, an indicator of microglia activation, was examined after mFPI in the mouse using Iba-1 immunohistochemistry. Iba-1 labels all microglia, however, tissue from a 6 hr sham (40×) (B) compared to a 6 hr mild injury (40×) (C) and a 6 hr moderate injury (40×) (D) show distinct differences in microglia morphology. Microglia in sham (B) demonstrated thin ramified processes (denoted by arrows) strongly contrasting the larger cell bodies and thicker processes (denoted by arrowheads) characteristic of activated microglia observed in the diffuse injured mouse (C, D).

Following brain injury there is an up-regulation of pro-inflammatory cytokines [30] that include IL-1β [12], [18]. IL-1β is a cytokine with sleep regulatory substance activity [10], [16], [31] which could partially explain post-traumatic sleep. A temporal profile of IL-1β indicated that cortical levels increased rapidly following moderate injury as compared to uninjured sham (Figure 6A). Levels of IL-1β peak at or near 9 hours post-injury and return to baseline levels by 12 hours post-injury. There was a significant increase in IL-1β levels in brain-injured animals compared to sham (F(7,21) = 6.474, p = 0.0004) and selected comparisons using the Bonferroni post-hoc analysis indicated a significant increase between sham and brain-injured mice at 1, 3 and 9 hours post-injury.

Microglia morphology, an indicator of microglia activation, was examined after diffuse brain injury in the mouse using Iba-1 immunohistochemistry. Iba-1 labels all microglia, however, distinct morphological differences in Iba-1 stained microglia were observed in brain-injured (mild and moderate injury, 09:00, Figure 6 C,D) compared to uninjured (Figure 6B) cortex at 6 hours post-injury. Microglia in brain-injured cortex showed morphologies consistent with activated microglia, including amoeboid cell bodies with thick, densely labeled processes (denoted by arrowheads). In contrast, thin, highly ramified processes of ramified microglia (denoted by arrows) were present in the uninjured sham cortex.

## Discussion

Brain injury survivors report varying degrees of sleep disturbances [32], however, the contribution of acute post-traumatic sleep to the injury itself remains unclear. To achieve this long-term goal, we undertook the present study to measure the acute sleep response to diffuse TBI, which we term post-traumatic sleep. We chose to focus on acute sleep post-injury, because sleep itself may be restorative and aid in the recovery of function following injury. By non-invasively recording sleep immediately following diffuse brain injury, we were able to document the induction of post-traumatic sleep. Altogether, our data, for the first time, support the hypothesis that diffuse brain injury promotes acute post-traumatic sleep in the mouse, and secondary injury related cellular processes coincide with this increase.

### Acute sleep measured using novel non-invasive cage system

Current sleep research associated with TBI has focused on chronic sleep disorders in the sequelae of human injury [32]–[34]. The lack of studies investigating acute sleep following TBI heightens the importance of studies in this field, because evidence promoting or disrupting sleep after TBI may change the standard of care for brain-injury patients. By investigating the role of post-traumatic sleep, interventions can be developed to mitigate damage. This study used a non-invasive sleep monitoring cage system which reliably measures injury-induced alterations in sleep [25] immediately following injury. Without the surgical procedures required for EEG recordings, we avoided the contraindications of an electrode in the brain at the time of injury which would create contusion or cavitation and most importantly allowed for post-injury sleep to be measured within minutes of the initial injury. The piezoelectric sensor cages, in conjunction with computer algorithms, recorded the sleep of each individual mouse (injury and sham) and created a detailed sleep profile that included mean percent sleep and median bout length. The cage system allows for sleep profiles of brain-injured and sham mice to be measured in exactly the same way. We found that post-traumatic sleep was significantly increased after brain injury, regardless of time of day or injury severity, with longer median sleep bouts underlying the overall increased post-traumatic sleep.

Despite the limitation of not being able to discriminate stages of sleep (rapid-eye-movement-sleep, slow wave sleep), the piezoelectric cage system is capable of accurately distinguishing wake from sleep [26]. While the sleep cage system has been well validated in distinguishing sleep from wake with comparisons between EEG/EMG and human observation in normal mice [25], it is possible that sleep could be over-estimated in TBI mice. If this were true, we would expect percent sleep times to be greatest in the first hour after injury and for moderate TBI to show a greater sleep increase than mild TBI. Since TBI mice were able to make postural adjustments and voluntary movements that signal wake, post-traumatic sleep is likely to be sleep, rather than a more severe condition. While the post-traumatic sleep bears many hallmarks of normal sleep, we cannot rule out that the increased sleep time is in part due to non-convulsive seizures with behavioral and temporal dynamics similar to sleep. We discount non-convulsive status epilepticus (NCSE) as a component of post-traumatic sleep because the development of epilepsy following experimental brain injury does not occur within the first week post-injury [35]. Following severe lateral fluid percussion in rats, EEG recordings indicated no injury-induced seizure within the first six hours post-injury [36]. Furthermore, EEG/EMG signal features used to determine sleep stages in normal mice may be compromised after brain injury and affect sleep-wake scoring. It should also be noted EEG/EMG recordings can result in false positive or negative sleep determination leading to possible error when used as the sole arbiter of sleep. For the purposes of this study, sleep determination did not rely fundamentally on EEG/EMG measures, but more simply is associated with a reversible perceptual disengagement from the environment, marked by a suspension of voluntary bodily functions. Ongoing work with the sleep cage system suggests that signal processing can distinguish REM from NREM sleep based on more irregular breathing during REM stages. Future work will determine whether the increases we observed in post-traumatic sleep arise from increases in REM or NREM sleep, or most likely from increases in both.

### Acute post-traumatic sleep increased regardless of time of injury

In order to explore the impact of diffuse TBI on natural sleep, mice were subjected to injury at two time points in their circadian rhythms. By conducting the injuries at the light/dark transition, we investigated whether post-traumatic sleep was a result of the brain injury or an interaction with natural biological tendencies to sleep. The 9:00 time point followed the onset of the light cycle, a time when nocturnal mice were expected to sleep. The 21:00 time point followed the onset of the dark cycle, a time when nocturnal mice are most active. Sleep patterns of uninjured mice showed circadian related pressures. Acute post-traumatic sleep significantly increased in comparison to the sleep of uninjured shams independent of the time of day mice were subjected to injury. This degree of increase in sleep following TBI is similar to the mean percent sleep of mice following 6 hours of sleep deprivation [37]. It is possible that the circadian clock itself or its outputs are dysregulated by TBI [34], which would contribute to injury-induced sleep being independent of the time at which the injury occurs. However, immediate permanent pathology is unlikely, as sleep parameters return to sham levels beyond 6 hours post-injury.

### Acute post-traumatic sleep increased independent of injury severity

We also examined the relationship between injury severity and post-traumatic sleep. Severity of the initial injury is considered a major determining factor for magnitude of secondary injury processes and outcome following TBI [38], which led to the hypothesis that injury severity would directly impact post-traumatic sleep. Contrary to our hypothesis, both mildly and moderately brain-injured mice showed similar significant increases in post-traumatic sleep compared to sham values. Even mild injury significantly increased sleep, which urges continued investigation into the contribution of post-traumatic sleep to the natural course of the injury. The possibility exists to induce an even milder injury, which may have less impact on post-traumatic sleep; however this may reduce/eliminate all other cellular hallmarks of TBI as well. Unfortunately, because mildly and moderately injured mice have comparable increases in acute post-traumatic sleep, the utility of sleep to serve as a diagnostic biomarker for injury severity is limited.

Our data also exclude the possibility of an injury-induced coma, as an extreme manifestation of sleep. Severe TBI can lead to coma, however, our brain-injured mice exhibit a brief period of non-responsiveness measured by the suppression of the righting reflex. Also, the maximum median bout length, measured in seconds, was 30 seconds, followed by periods of wake activity, which excludes the possibility of an injury-induced coma since mice voluntarily woke between sleep bouts. These periods of wake activity during the interbout interval between sleep activity were clearly shown by the piezoelectric sensor data.

### Inflammation as a secondary injury mechanism associated with overall increases in acute post-traumatic sleep

TBI is characterized by two pathological phases: cellular injury resulting from primary impact and the ensuing secondary injury mediated by pathological processes [17]. Secondary injury occurs over time post-injury with a more gradual onset beginning minutes to hours after impact and contributes to the clinical morbidities associated with TBI. Post-traumatic sleep in 5 minute intervals showed that the increase in mean percent sleep over the first hour post-injury is time dependent (Figure 2B). If the primary impact solely contributed to post-traumatic sleep, then an immediate increase in post-traumatic sleep to a maximum level would have been observed. The secondary injury cascades that play a role in inducing sleep following diffuse TBI likely include post-traumatic signaling that activate glia, as evidenced by increased production of pro-inflammatory cytokines, such as IL-1β, in both animal models and human head injury patients [18], [30].

Activated microglia can contribute to the production of IL-1β after TBI. Once produced, IL-1β acts locally to affect neuronal assemblies, altering their functional status, as well as acting on sleep regulatory circuits [11]. Our group has accumulated evidence for circuit disruption, dismantling and reorganization in the diffuse-injured cortex, particularly the whisker sensory circuit of the rat [39]–[43]. Microglia likely act as the effectors of circuit disruption [40] by producing cytokines and as a consequence influence the functional state of those circuits. The impact of microglia can then extend beyond local circuits to sleep regulatory circuits and ultimately induce sleep [10], [16], [44]. In the injured mouse brain, these microglial signaling processes that influence sleep last 6 hours post-injury and remain to be determined in the human condition.

Our data show that IL-1β was upregulated in the cortex following diffuse TBI, and previous studies have reported that humans undergoing IL-1β therapy report excessive sleepiness [11]. Injections of IL-1β enhance NREM sleep [16] and application of IL-1β to the somatosensory cortex leads to enhanced EEG delta wave activity [45]. These data indicate a mechanistic link between IL-1β and sleep. In the injured cortex, IL-1β continues to increase, peaking at or near 9 hours post-injury and returning to sham levels by 12 hours post-injury (Figure 6A), similar to the increase in mean percent sleep in brain-injured mice. Collectively, these data suggest that post-traumatic sleep may involve inflammatory mediated processes and the upregulation of pro-inflammatory cytokines that can act as sleep regulatory substances. Our immunohistological staining indicated activation of microglia in the cortex of mild and moderate brain-injured mice compared to uninjured sham at 6 hours post-injury, coinciding with elevated cytokine levels in moderate injury and the end of post-traumatic sleep. Activated microglia produce pro-inflammatory cytokines, including those with dual roles as SRSs (IL-1β, IL-6, TNFα) [46], as indicated by pharmacological inhibition of microglia reducing levels of pro-inflammatory cytokines. The infiltration and activation of microglia may be a potential source of sleep regulatory factors in the injured brain [40], [47], [48], regardless of injury severity. We argue that increases in sleep following TBI may result from the inflammatory response associated with the secondary injury in which elevated cytokine levels are associated with activation of microglia after brain injury. Future studies are needed to examine the mechanistic relationship between changes in cytokine levels and sleep.

## Conclusion

The current study demonstrated that acute sleep was increased following diffuse TBI, and injury-induced cellular cascades may contribute to this increase. The increase in sleep was independent of time of day that the injury occurred and the injury severity. Increases in median bout length contributed to the overall increase in sleep observed post-injury. Further studies need to determine the cellular benefit or detriment of acute post-traumatic sleep on recovery following TBI (and other neurological conditions) by disrupting acute sleep. Understanding the role of acute post-traumatic sleep on outcome can begin to answer the controversial question, “Should one sleep, be frequently awoken or left uninterrupted after a concussion?”
